# The complete chloroplast genome of *Nomocharis pardanthina*

**DOI:** 10.1080/23802359.2018.1424581

**Published:** 2018-01-09

**Authors:** Hai-Ying Liu, Juan Li, Deng-Feng Xie, Xing-Jin He, Yan Yu, Song-Dong Zhou

**Affiliations:** Key Laboratory of Bio-Resource and Eco-Environment of Ministry of Education, College of Life Sciences, Sichuan University, Chengdu, P.R.China

**Keywords:** Chloroplast, genome, *Nomocharis*, *Lilium*

## Abstract

*Nomocharis pardanthina* Franchet (Liliaceae) is an endangered species naturally distributed in China. The complete chloroplast genome sequence of *N. pardanthina* was generated by de novo assembly using whole genome next generation sequencing data. The chloroplast genome of *N. pardanthina* was 152,718 bp in length and divided into four distinct regions, such as large single copy region (82,154 bp), small single copy region (17,524 bp) and a pair of inverted repeat regions (26,520 bp). The genome annotation predicted a total of 112 genes, including 78 protein-coding genes, 30 tRNA genes, and 4 rRNA genes. Phylogenetic analysis with the reported chloroplast genomes revealed that *N. pardanthina* is nested in *Lilium* and has close relationship to *L. bakerianum* and *L. taliense*.

*Nomocharis pardanthina* Franchet (Liliaceae) is an herbaceous plant species naturally distributed in China. It wildly grows in forest margins and grassy slopes at an elevation of 2700–4100 m (Liang and Tamura 2000). *Nomocharis pardanthina* shows unique flower morphology different from other species in Liliaceae and is placed in the genus *Nomocharis* Franchet. However, recent molecular phylogenetic analysis indicates that *Nomocharis* is nested within *Lilium* (Hayashi and Kawano 2000; Rønsted et al. 2005; Gao et al. 2012, 2013). Although several phylogenetic studies reported nuclear and chloroplast sequences of *N. pardanthina*, the complete chloroplast genome sequence is not available till now. Here, we report the complete chloroplast genome sequence of *N. pardanthina* to provide a genomic resource and to clarify phylogenetic relationship of this plant with other species in the Liliaceae family.

Total genomic DNA was isolated from mature leaves sampled from Mt. Gaoligong (27°47′23″N 98°27′04″E), Yunnan Province, China. Voucher specimens were deposited in SZ (Sichuan University Herbarium). Total genomic DNA was extracted by Plant Genomic DNA Kit. The isolated genomic was manufactured to average 350 bp paired-end(PE) library using Illumina Hiseq platform (Illumina, San Diego, CA), and sequenced by Illumina genome analyser (Hiseq PE150). Owing to the inference of nuclear genome, chloroplast genome-related reads were sieved by mapping to the closer species *L. taliense*. Contigs, assembled using SOAPdenovo2 (Luo et al. 2012), were sorted and joined into a single-draft sequence using Geneious (Kearse et al. 2012), by comparison with the chloroplast sequence of *L. taliense* as a reference. Gapcloser was used to fill the gapped sites, and the draft sequence was corrected manually by clean read mapping using bowtie2 (Langmead and Salzberg 2012) and Tablet (Milne et al. 2013). The genes in chloroplast genome were predicted using Geneious and corrected manually.

The complete chloroplast genome of *N. pardanthina* (GenBank accession no. MG704135) was a circular form of 152,718 bp in length, which was separated into four distinct regions such as large single copy (LSC) region of 82,154 bp, small single copy (SSC) region of 17,524 bp and a pair of inverted repeat regions of 26,520 bp. Overall GC contents of chloroplast genomes were 37.00%. The chloroplast genome contained a total of 112 genes including 78 protein-coding genes, 30 tRNA genes, and 4 rRNA genes.

In order to understand the phylogenetic relationship between *N. pardanthina* and related species, the complete chloroplast genome sequence of 16 genera (24 species) were aligned by MAFFT (Katoh et al. 2002) and trimmed by trimAl (Capella-Gutierrez et al. 2009). The evolution history was inferred by using maximum-likelihood method based on Tamura-Nei model in MAGA7.0 (Kumar et al. 2016). Bootstrap (BS) value was calculated from 1000 replicate analysis. The phylogenetic tree was divided into seven independent groups, which were Amaryllidaceae, Campynemataceae, Alstroemeriaceae, Colchicaceae, Melanthiaceae, Smilacaceae, and Liliaceae (Figure 1). As was expected, *N. pardanthina* was closely grouped with the genus *Lilium*, and occurred as a sister group with clade of *L. bakerianum* and *L. taliense* with 100% BS value.

**Figure 1.**
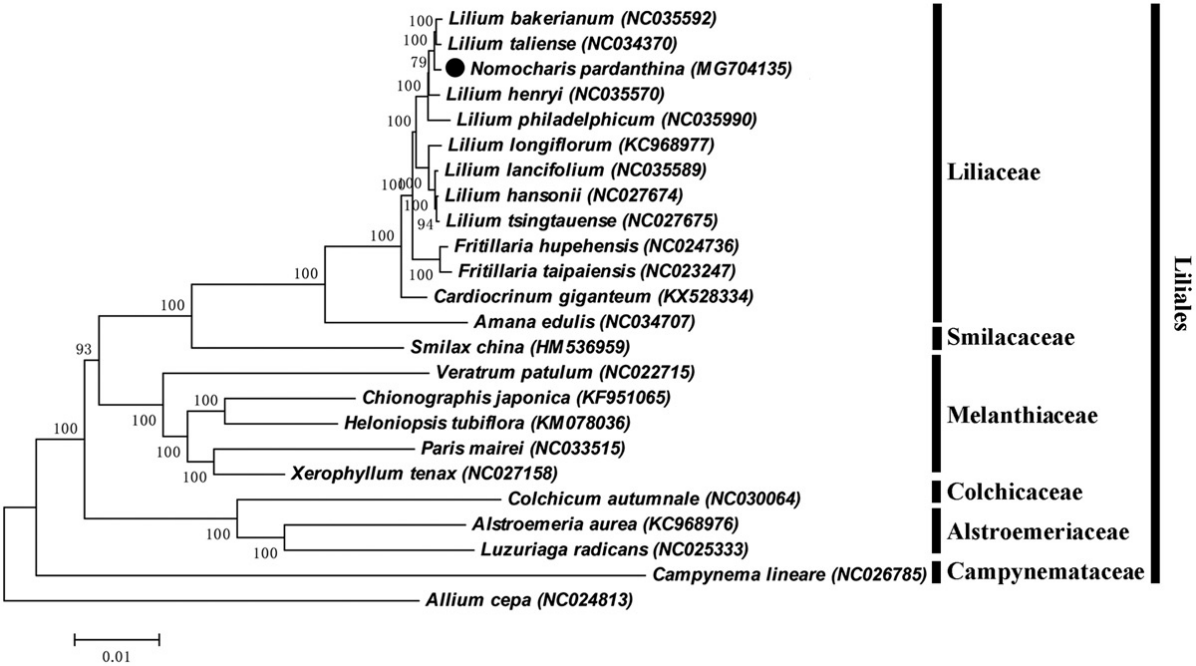
ML phylogenetic tree of *N. pardanthina* with 22 species in the Liliales order was constructed by chloroplast genome sequences. Numbers on the nodes are bootstrap values from 1000 replicates. *Allium cepa* was selected as an outgroup.
